# Tobacco smoking and the risk of aortic dissection in the UK Biobank and a meta-analysis of prospective studies

**DOI:** 10.1038/s41598-025-96529-y

**Published:** 2025-04-09

**Authors:** Momina Yawar I. Khan, Alison Dillman, Marina Sanchez-Perez, Makoto Hibino, Dagfinn Aune

**Affiliations:** 1 Department of Epidemiology and Biostatistics, School of Public Health, Imperial College London, White City Campus, 90 Wood Lane, London, W12 0BZ UK; 2 Department of Thoracic and Cardiovascular Surgery, Heart, Vascular, and Thoracic Institute, Cleveland Clinic, Cleveland, OH USA; 3 Department of Nutrition, Oslo New University College, Oslo, Norway; 4 Department of Research, Cancer Registry of Norway, Norwegian Institute of Public Health, Oslo, Norway

**Keywords:** Tobacco, Smoking, Aortic dissection, Cohort, Meta-analysis, Cardiology, Diseases, Risk factors

## Abstract

Tobacco smoking is thought to increase risk of aortic dissection, however, few prospective studies have been published on the association to date and little is known about the effect of smoking cessation on risk of aortic dissection. To clarify these associations we investigated the association between different aspects of tobacco smoking and aortic dissection in the UK Biobank Study and we summarized the available data in a meta-analysis of cohort studies. Multivariable Cox proportional hazards models were used to estimate hazard ratios (HRs) and 95% confidence intervals (CIs) for the association between smoking and risk of aortic dissection in the UK Biobank. PubMed and Embase were searched up to 19th of July 2024 to identify relevant cohort studies and random effects models were used to calculate summary relative risks (RRs) and 95% CIs in the meta-analysis. During a mean follow-up of 12.3 years a total of 376 incident cases of aortic dissection were identified among 499,078 participants. The multivariable adjusted hazard ratio for current and former smokers versus never smokers were 2.48 (1.87–3.29) and 1.03 (0.81–1.29), respectively. There was a dose-response between increasing number of cigarettes smoked per day and aortic dissection risk with HRs (95% CIs) of 2.31 (1.13–4.71), 2.94 (1.88–4.58), and 2.63 (1.65–4.37) for 1–9, 10–19, and ≥ 20 cigarettes/day among current smokers. Greater number of pack-years was positively associated with aortic dissection (1.66, 1.21–2.28 for ≥ 30 pack-years vs. never smokers). Former smokers who had quit for varying durations had a 48-75% reduction in aortic dissection risk when compared to current smokers. In the meta-analysis, the summary RR was 2.44 (1.65–3.60, I^2^ = 68%, *n* = 3) for current and 1.32 (0.72–2.40, I^2^ = 75%, *n* = 3) for former vs. never smokers, 1.52 (1.30–1.79, I^2^ = 31%, *n* = 3) per 10 cigarettes/day, 1.16 (1.06–1.28, I^2^ = 44%, *n* = 3) per 10 pack-years, and 0.78 (0.71–0.86, I^2^ = 0%, *n* = 2) per 10 years since quitting smoking. These results provide further support that tobacco smoking increases the risk of developing aortic dissection with some indication of a dose-response with increasing intensity and amount, while smoking cessation attenuated this risk. Although further studies are needed, these findings provide further support for policies to reduce the prevalence of smoking in the general population and to promote smoking cessation among smokers.

**Supplementary Information:**

The online version contains supplementary material available at 10.1038/s41598-025-96529-y.

## Introduction

Aortic dissection is a relatively uncommon, but highly fatal disease with an incidence of 3–6 cases per 100,000 persons per year in Europe and America^1^. Aortic dissection can lead to organ damage including kidney failure and intestinal damage, stroke, aortic valve damage and cardiac tamponade, which can end in disability and/or death^1^. Although rapid diagnosis and access to treatment is of major importance for the prognosis in aortic dissection, severe complications are not uncommon^1^. Primary prevention may therefore be of major importance in reducing its public health impact, however, relatively few modifiable risk factors have been established. Hypertension, and particularly elevated diastolic blood pressure is a risk factor for aortic dissection^2–4^. In addition, there is some suggestion that higher apoA1^3^ and a history of diabetes mellitus^4–6^ is associated with reduced risk.

Smoking is one of the leading modifiable risk factors for a wide range of cardiovascular diseases (CVDs) including coronary heart disease, stroke, heart failure, atrial fibrillation, sudden cardiac death and abdominal aortic aneurysms^7–11^. There is some evidence that tobacco smoking increases the risk of developing aortic dissection^3,4,12–15^, however, relatively few cohort studies have been published to date. In the Japan Collaborative Cohort study (91141 participants, 110 AD deaths, 16.4 years of follow-up) a 2.7-4 fold increase in aortic dissection mortality was observed among current smokers vs. never smokers^12^. In the Ibaraki Prefectural Health Study (95723 participants, 188 aortic dissection deaths, 26 years follow-up) current smokers who smoked ≥ 20 cigarettes/day had a 2.4-fold increase risk in aortic dissection mortality compared to never smokers^4^. In the Malmö Diet and Cancer Study (30,412 participants, 70 cases, 16 years follow-up)^3^ a two-fold increase in risk of aortic dissection was observed among current smokers/former smokers who had quit within the past year vs. never/former smokers who had quit > 1 year ago, and it was estimated that smoking accounted for 14% of the cases. In the Honolulu Heart Program (7682 Japanese American men, 23 cases, 20 years follow-up), a non-significant positive association was observed with a hazard ratio of 1.68 (95% CI: 0.67–4.18) per 62 cigarette pack-years^14^. In the Whitehall study (18,000 male participants, 38 aortic dissection deaths, 18 years follow-up), there was a 7.7-fold increase in risk for current vs. non-current smokers^13^. Lastly, a Japanese case-control study found a 3.5-fold increased odds of developing aortic dissection among current vs. non-current smokers^15^. Of the studies that looked at former smokers, one study reported increased risk among former versus never smokers^12^, however, another study reported no association^4^. Interestingly longer duration of smoking cessation was associated with lower risk in one study^12^, however, other studies have not investigated the impact of duration of smoking cessation on aortic dissection risk.

Given the limited number of published prospective cohort studies on tobacco smoking and risk of aortic dissection we therefore investigated this association further in the UK Biobank study, a large-scale prospective study of 0.5 million British participants. We particularly wanted to investigate different measures of smoking in more detail than what has previously been studied, including smoking status, cigarettes per day, duration of smoking, pack-years of smoking, age at starting smoking and time since quitting smoking in relation to risk of aortic dissection.

## Methods

### The UK Viobank study

The UK Biobank Study is a prospective study of 502,359 individuals aged 37–73 years at recruitment between 2006 and 2010^16^. Nine million people who were registered with the UK NHS were invited to partake in the UK Biobank study and the response rate was 5.5%. Participation was voluntary and all participants provided written informed consent. Data was collected on diet, smoking, alcohol intake, socio-demographic characteristics, previous and current medical history via touchscreen questionnaires (36). In the current analysis, we excluded in subsequent order persons with prevalent aortic dissection at baseline (*n* = 62), different self-reported compared to genetic sex (*n* = 372), and missing information on smoking status (*n* = 2847), leaving 499,078 participants included in the primary analysis. In sensitivity analyses, further exclusions were made for persons with prevalent ischemic heart disease, stroke, cancer (except non-melanoma skin cancer), or respiratory disease at baseline (*n* = 138809), leaving 360,259 participants for those analyses.

### Exposure assessment

Detailed data on smoking, alcohol intake, socio-demographic characteristics, and medical history have been collected from the participants using touchscreen questionnaires, while anthropometric data such as weight, height, waist and hip circumference have been measured. Physical measurements as well as blood, urine and saliva samples were also taken at baseline in 2006 to be assessed for future analysis. For the current analysis, we used information on smoking status, number of cigarettes smoked per day, the age at which smoking started, duration of smoking, pack-years of smoking and time since individuals quit smoking. Participants with missing values on the smoking variables of interest were excluded from the respective analyses.

### Outcome assessment

Incident cases of aortic dissection were identified by linkages to Hospital Episode Statistics for England (HES), Scottish Morbidity Record (SMR), and the Patient Episode Database for Wales (PEDW). Participants with aortic dissection listed as underlying cause of death were identified by linkages to NHS England (for England and Wales) and NHS Central Register, National Records of Scotland. Aortic dissection cases were identified according to the 10th revision of the International Statistical Classification of Diseases (ICD-10) diagnostic code I71.0 and ICD-9 code 441.0. For participants with aortic dissection listed as underlying cause of death, but with no prior hospital diagnosis of aortic dissection, we used their date of death as diagnosis date, while for hospitalizations we used the date of hospital diagnosis as diagnosis date. Participants were followed up from the date of recruitment at baseline and were censored at the date of aortic dissection diagnosis, death, loss to follow-up, or the last complete follow-up (30 September 2021 for England and Wales and 31 October 2021 for Scotland), whichever occurred first. Loss to follow-up was low with 1275 participants (0.26%) lost over the study period.

### Statistical analysis

#### UK biobank

Multivariable Cox proportional hazards regression models were used to estimate hazard ratios (HRs) and 95% confidence intervals (CIs) for the association between tobacco smoking and risk of developing aortic dissection. The proportional hazards assumption was tested using Schoenfeld residuals and there was no evidence of violation of the proportional hazards assumption (*p* > 0.05). The multivariable model included age (continuous), sex (male vs. female), ethnicity (white and non-white), Townsend deprivation index (quintiles), education (quintiles), body mass index (BMI) (categorical), height (sex-specific quintiles), total physical activity (quintiles), history of connective tissue disease (Marfan syndrome or Ehlers-Danlos syndrome) (yes vs. no), and each smoking variable (categorical). Missing data on the confounders of interest was in general low (0.12-1.8%) with the exception of physical activity (8.8%), and were handled using a missing category indicator. We did not include hypertension or other circulatory disorders as covariates in the main model because there is evidence that smoking may increase risk of hypertension^17,18^ and a wide range of circulatory diseases^7^, and these could therefore be on the biological pathway to aortic dissection development, but we conducted a sensitivity analysis with further adjustment for hypertension status.

Sensitivity analyses were conducted excluding participants with ischemic heart disease, stroke, cancer (except non-melanoma skin cancer), and respiratory disease at baseline and additionally excluding the first five years of follow up, to take into account potential reverse causation. Subgroup analyses stratified by age, sex, hypertension and BMI were conducted to investigate potential effect modification. A Wald test was used to test for interactions.

#### Meta-analysis

PubMed and Embase databases were searched up to 19th of July 2024 for relevant cohort studies on smoking and aortic dissection. Prospective cohort, retrospective cohort, case cohort, and nested case-control studies within cohort studies that reported adjusted risk estimates and 95% confidence intervals for the association between smoking and aortic dissection risk were eligible for inclusion. Information on first author, publication year, geographic location, study name, study period, number of participants, age, sex, and number of cases or deaths, smoking exposure and comparison, risk estimates (95% CIs), and adjustment for confounders were extracted into a table. Random effects models were used to estimate summary relative risks (RRs) and 95% confidence intervals (CIs) for the association between different measures of smoking and aortic dissection risk^19^. Linear dose-response analyses were conducted to estimate the association between increasing number of cigarettes per day, pack-years and duration of smoking cessation the risk of aortic dissection using the method by Greenland and Longnecker^20^. For smoking variables that were reported in ranges (cigarettes/day, pack-years, years since quitting smoking), we used the average of the lower and upper cut-off point to estimate a midpoint for each category, unless the mean/median per category was reported directly. Heterogeneity between studies was assessed using I^2^-statistics^21^. The computer program Stata 16.1 (College Stations, TX, USA) was used for all statistical analyses and a two-sided p-value < 0.05 was considered statistically significant.

## Results

### UK biobank

The current analysis included 499,078 participants (227400 men, 271678 women) with a mean [median] age of 56.5 [58] years at baseline. Table 1 shows the baseline characteristics for various variables by smoking status (never, former, and current smokers). Current smokers were younger than former and never smokers. More men than women identified themselves as current smokers than never smokers (Table 1). Small differences were observed in smoking status when stratified by ethnicity. Current smokers had higher deprivation scores, and lower education. There was little difference in BMI across smoking status categories, except for a higher BMI among former smokers, and there was no meaningful differences in height, total physical activity, or prevalence of connective tissue disease (Marfan’s syndrome or Ehlers-Danlos syndrome). The prevalence of hypertension and systolic blood pressure was higher in former smokers than in never smokers.

**Table 1:** Baseline characteristics according to smoking status.

		Smoking status		
		Never	Former	Current
Participants (n)		273,254	172,945	52,879
Age (years)		57	60	55
Sex (%)	Men	40.7	50.6	54.0
	Women	59.3	49.4	46.0
Ethnicity (%)	White	92.9	96.8	93.5
	Non-white	6.8	2.9	6.1
	Missing	0.3	0.3	0.4
Townsend deprivation index (%)	Quintile 5 (most deprived)	16.7	19.8	36.2
Education (%)	University	35.6	29.5	22.7
BMI (median), kg/m^2^		26.4	27.3	26.5
Height, men/women (median), cm		176/162	175/163	175/162
Hypertension (%)		52.9	59.6	50.8
Systolic blood pressure (median), mmHg		135.5	138	133.5
Diastolic blood pressure (median), mmHg		82	82	81
Total physical activity frequency per week (median)		11	11	11
History of connective tissue disease (%)^1^		0.05	0.05	0.05

^1^Marfan’s syndrome or Ehlers-Danlos syndrome.

During a mean follow-up of 12.3 years (6140424 person-years) a total of 376 cases of aortic dissection occurred. The HR (95% CI) for developing aortic dissection was 2.48 (95% CI: 1.87–3.29) for current smokers and 1.03 (0.81–1.29) for former smokers when compared to never smokers after adjustment for age, sex, ethnicity, education, Townsend deprivation index, BMI, height, physical activity, and history of connective tissue disease.

There was indication of a dose-response relationship between increasing number of cigarettes smoked per day and aortic dissection risk, with HRs (95% CIs) of 2.31 (1.13–4.71), 2.94 (1.88–4.58), and 2.63 (1.65–4.37, p_trend_<0.001) for 1–9, 10–19, and ≥ 20 cigarettes/day among current smokers with never smokers as reference category (Table 2). There was also a significant association between pack-years smoked (≥ 30 vs. never), and duration of smoking (≥ 40 years vs. never) among current smokers and aortic dissection incidence and the HR (95% CI) were 1.66 (1.21–2.28, p_trend_=0.002) and 2.19 (1.46–3.30, p_trend_<0.001), respectively (Table 2). Younger age at starting smoking was not more strongly associated with aortic dissection than older age and the HRs were 1.88 (95% CI: 1.05–3.35) for ≤ 15 years vs. 3.07 (2.18–4.33) for ≥ 16 years, when compared to never smokers (Table 2). The HRs were 0.52 (0.34–0.82), 0.38 (0.23–0.62), 0.25 (0.14–0.43), 0.49 (0.32–0.74) for < 10, 10-<20, 20-<30, and ≥ 30 years of smoking cessation vs. current smokers and these estimates were comparable to never smokers (0.40, 0.30–0.53) (Table 2).

**Table 2:** Hazard ratios (95% CIs) of aortic dissection incidence according to smoking variables.

Smoking status		Never	Former	Current	Ever			p_trend_
	Participants	273,254	172,945	52,879	225,824			
	Person-years	339,1748.3	2,112,828.2	635,847.7	2,748,675.9			
	Cases	165	137	74	211			
	HR (95% CI)^1^	1.00	1.03 (0.81–1.29)	2.48 (1.87–3.29)	1.29 (1.05–1.59)			
Cigarettes/day		Never	Former	< 10 cig/d (5)	10-<20 (14)	≥ 20 (20)		
	Participants	273,254	172,945	7207	15,405	13,477		
	Person-years	3,391,748.3	2,112,828.2	87,670.2	185,514.7	158,659.7		
	Cases	165	137	8	23	19		
	HR (95% CI)^1^	1.00	1.03 (0.81–1.29)	2.31 (1.13–4.71)	2.94 (1.88–4.58)	2.63 (1.65–4.37)		< 0.001
Pack-years		Never	< 10 (5.75)	10-<20 (14.53)	20-<30 (24.25)	≥ 30 (40.8)		
	Participants	273,254	37,614	40,607	29,343	43,168		
	Person-years	3,391,748.3	464,066.1	497,812.7	356,775.1	505,817.5		
	Cases	165	30	31	27	58		
	HR (95% CI)^1^	1.00	1.27 (0.86–1.87)	1.12 (0.76–1.66)	1.30 (0.86–1.96)	1.66 (1.21–2.28)		0.002
Duration of smoking among current smokers		Never	< 30 years (25)	30-<40 (35)	≥ 40 (45)			
	Participants	273,254	8527	13,439	16,722			
	Person-years	3,391,748.3	106,315.6	164,391.7	191,617.9			
	Cases	165	9	17	30			
	HR (95% CI)^1^	1.00	3.85 (1.89–7.87)	3.11 (1.84–5.25)	2.19 (1.46–3.30)			< 0.001
Duration of smoking among former smokers		Never	< 20 years (12)	20-<30 (24)	≥ 30 (37)			
	Participants	273,254	52,082	30,328	32,328			
	Person-years	3,391,748.3	642,146.6	370,545.7	382,788.6			
	Cases	165	39	14	41			
	HR (95% CI)^1^	1.00	1.01 (0.71–1.43)	0.59 (0.34–1.02)	1.41 (0.98–2.01)			0.38
Age started smoking		Never	Former, ≤ 15 years	Former, ≥ 16 years	Current, ≤ 15 years	Current, ≥ 16 years		
	Participants	273,254	33,930	81,268	13,204	25,484		
	Person-years	3,391,748.3	410,832.9	990,133.4	156,298.9	306,026.3		
	Cases	165	28	67	13	43		
	HR (95% CI)^1^	1.00	1.04 (0.69–1.57)	1.01 (0.75–1.34)	1.88 (1.05–3.35)	3.07 (2.18–4.33)		
Years since quitting		Current	< 10 years (4)	10-<20 (15)	20-<30 (25)	≥ 30 (34)	Never	
	Participants	52,879	31,565	26,828	30,788	26,088	273,254	
	Person-years	635,847.7	381,796.8	326,269.6	376,698.6	317,047.4	3,391,748.3	
	Cases	74	27	19	16	33	165	
	HR (95% CI)^1^	1.00	0.52 (0.34–0.82)	0.38 (0.23–0.62)	0.25 (0.14–0.43)	0.49 (0.32–0.74)	0.40 (0.30–0.53)	< 0.001

^1^Multivariable model adjusted for age, sex, ethnicity, Townsend deprivation index, education, BMI, height, total physical activity, history of connective tissue disease.

During 6141347.6 person-years of follow-up, 160 aortic dissection deaths occurred. Results for aortic dissection mortality were in general similar to those for incidence (Table 3).

**Table 3:** Hazard ratios (95% CIs) of aortic dissection mortality according to smoking variables.

Smoking status		Never	Former	Current	Ever			p_trend_
	Participants	273,254	172,945	52,879	225,824			
	Person-years	3,392,172.4	2,113,126.1	636,049.1	2,749,175.2			
	Deaths	70	63	27	90			
	HR (95% CI)^1^	1.00	1.14 (0.80–1.61)	2.32 (1.47–3.66)	1.34 (0.97–1.84)			
Cigarettes/day	Cut-off (median)	Never	Former	< 10 cig/d (5)	10-<20 (14)	≥ 20 (20)		
	Participants	273,254	172,945	7207	15,405	13,477		
	Person-years	3,392,172.4	2,113,126.1	87,699	185,570.3	158,720.1		
	Deaths	70	63	2	8	8		
	HR (95% CI)^1^	1.00	1.14 (0.81–1.62)	1.40 (0.34–5.72)	2.63 (1.25–5.52)	3.05 (1.44–6.46)		< 0.001
Pack-years	Cut-off (median)	Never	< 10 (5.75)	10-<20 (14.53)	20-<30 (24.25)	≥ 30 (40.8)		
	Participants	273,254	37,614	40,607	29,343	43,168		
	Person-years	3,392,172.4	464,124.5	497,883.9	356,848.2	505,965.7		
	Deaths	70	18	11	12	21		
	HR (95% CI)^1^	1.00	1.81 (1.08–3.04)	0.97 (0.51–1.83)	1.41 (0.76–2.61)	1.48 (0.89–2.47)		0.15
Duration of smoking among current smokers	Cut-off (median)	Never	< 30 years (25)	30-<40 (35)	≥ 40 (45)			
	Participants	273,254	8527	13,439	16,722			
	Person-years	3,392,172.4	106,332.9	164,435.6	191,707.5			
	Deaths	70	4	4	13			
	HR (95% CI)^1^	1.00	4.20 (1.43–12.31)	1.76 (0.62–4.97)	2.23 (1.20–4.15)			0.003
Duration of smoking among former smokers	Cut-off (median)	Never	< 20 years (12)	20-<30 (24)	≥ 30 (37)			
	Participants	273,254	52,082	30,328	32,313			
	Person-years	3,392,172.4	642,227	370,575	382,881.1			
	Deaths	70	22	8	15			
	HR (95% CI)^1^	1.00	1.36 (0.84–2.21)	0.81 (0.39–1.70)	1.22 (0.69–2.18)			0.61
Age started smoking	Cut-off	Never	Former, ≤ 15 years	Former, ≥ 16 years	Current, ≤ 15 years	Current, ≥ 16 years		
	Participants	273,254	33,930	81,268	13,204	25,484		
	Person-years	3,392,172.4	410,874.1	990,296.1	156,341.8	306,134.3		
	Deaths	70	11	34	7	14		
	HR (95% CI)^1^	1.00	1.09 (0.57–2.09)	1.22 (0.80–1.85)	2.85 (1.28–6.34)	2.55 (1.42–4.57)		
Years since quitting	Cut-off (median)	Current	< 10 years (4)	10-<20 (15)	20-<30 (25)	≥ 30 (34)	Never	
	Participants	52,879	31,565	26,828	30,788	26,088	273,254	
	Person-years	636,049.1	381,864.3	326,302.0	376,736.6	317,111.7	3,392,172.4	
	Deaths	27	9	9	7	21	70	
	HR (95% CI)^1^	1.00	0.46 (0.22–0.99)	0.47 (0.22-1.00)	0.28 (0.12–0.65)	0.80 (0.27–1.44)	0.43 (0.27–0.68)	0.28

^1^Multivariable model adjusted for age, sex, ethnicity, Townsend deprivation index, education, BMI, height, total physical activity, history of connective tissue disease.

### Sensitivity analyses

Further adjustment for hypertension status did not substantially alter these associations (Supplemental Tables 1 and 2). In sensitivity analyses excluding persons with prevalent ischemic heart disease, stroke, cancer, and respiratory disease at baseline, the association between smoking status and aortic dissection was slightly strengthened with HRs of 3.02 (2.12–4.30) for current smokers and 1.20 (0.89–1.69) for former smokers when compared to never smokers (Supplemental Table 3). Similarly, associations between cigarettes/day, pack-years and duration of smoking were also strengthened in these analyses (Supplemental Table 3), and similar results were observed for aortic dissection mortality (Supplemental Table 4). Additional exclusion of the first 5 years of follow-up did not materially alter these results further (Supplemental Tables 5 and 6).

In analyses stratified by age (*p* = 0.07), sex (*p* = 0.73), BMI (*p* = 0.44) and hypertension (*p* = 0.46) there were no significant interactions, although for age the p-value for interaction was approaching significance with a suggestive stronger association for current vs. never smokers (3.07, 2.14–4.41 vs. 1.77, 1.12–2.79) among participants aged ≥ 60 vs. those aged < 60 years (Supplemental Table 7).

### Meta-analysis

**Fig. 1:**
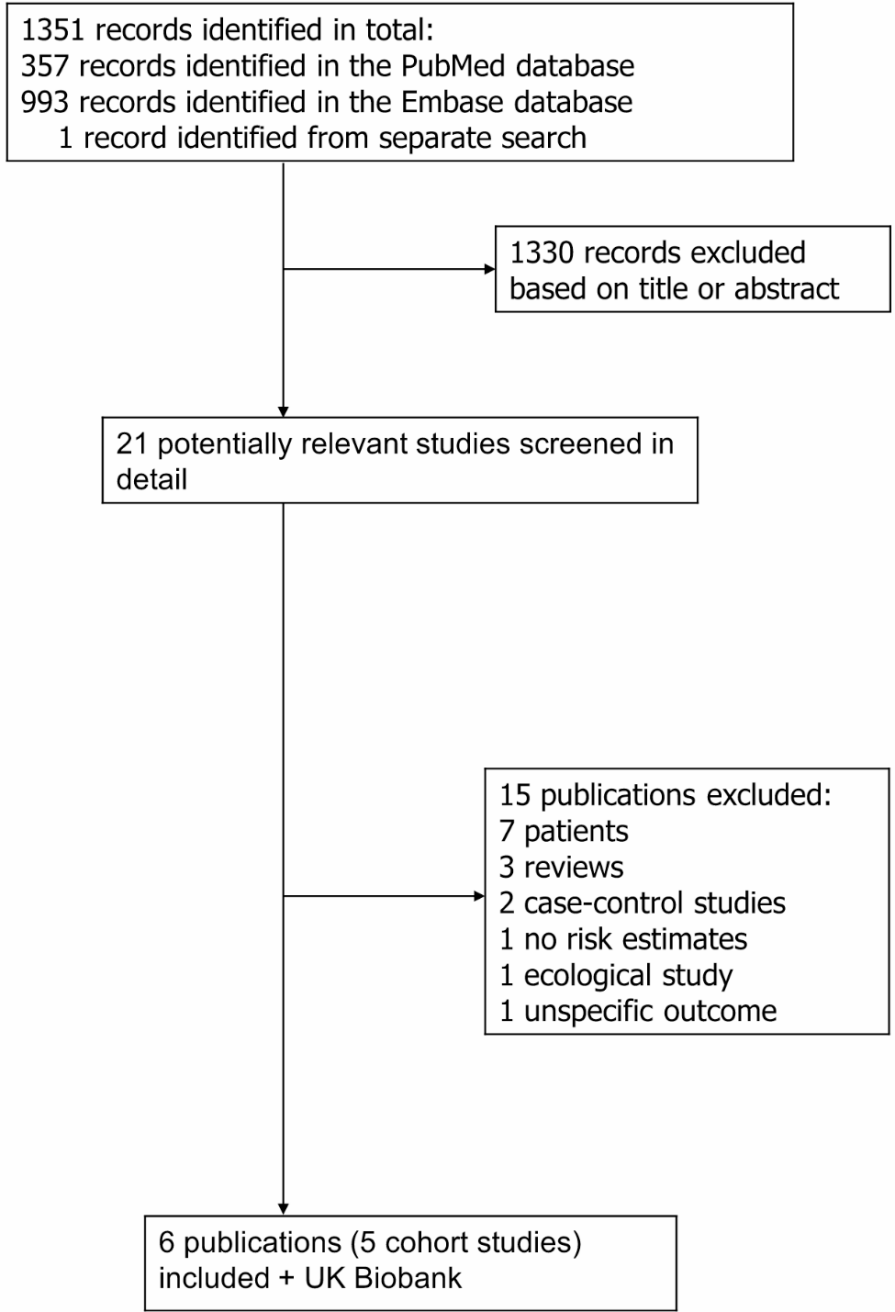
Flow-chart of study selection for meta-analysis.

**Fig. 2:**
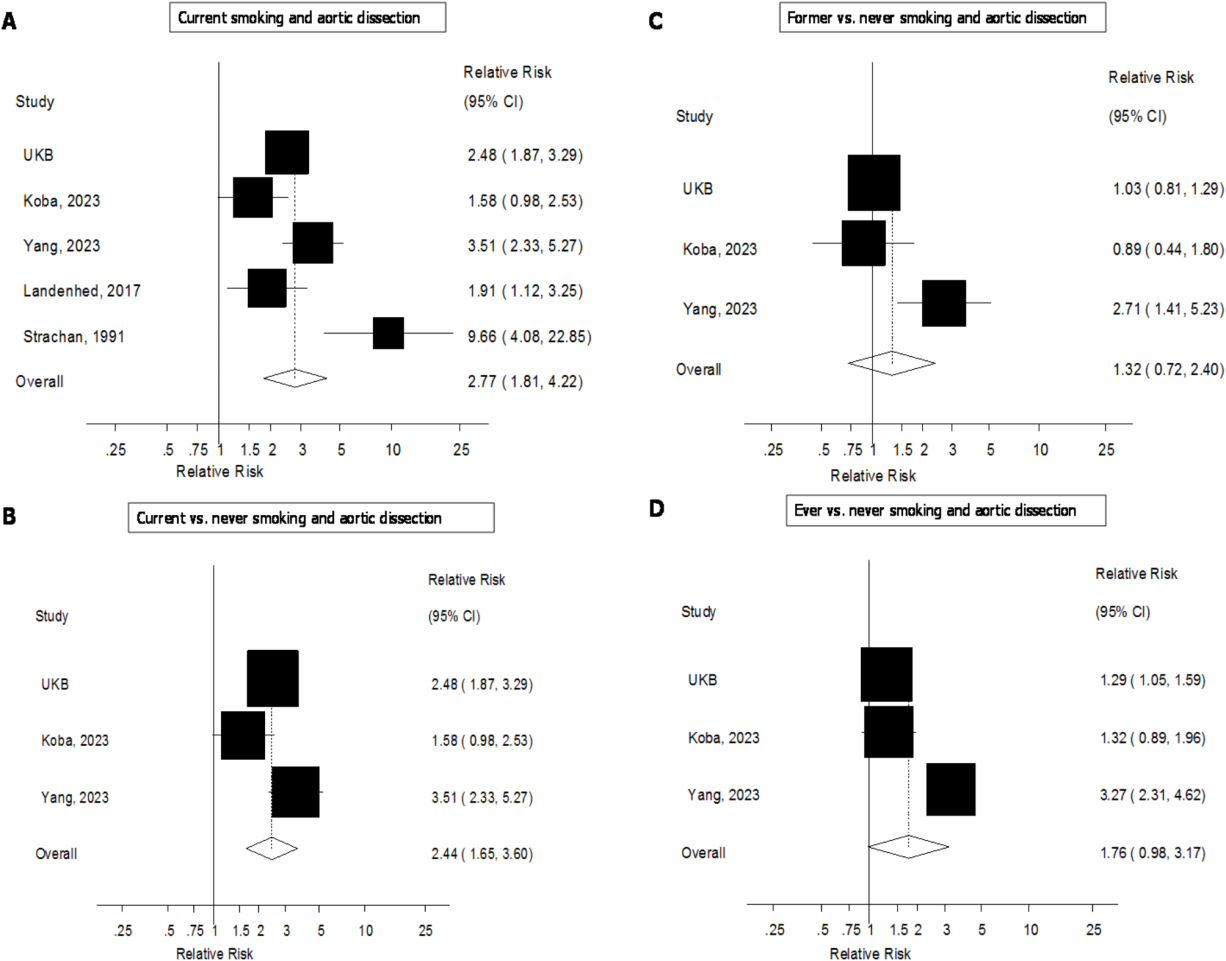
Smoking status and aortic dissection.

**Fig. 3:**
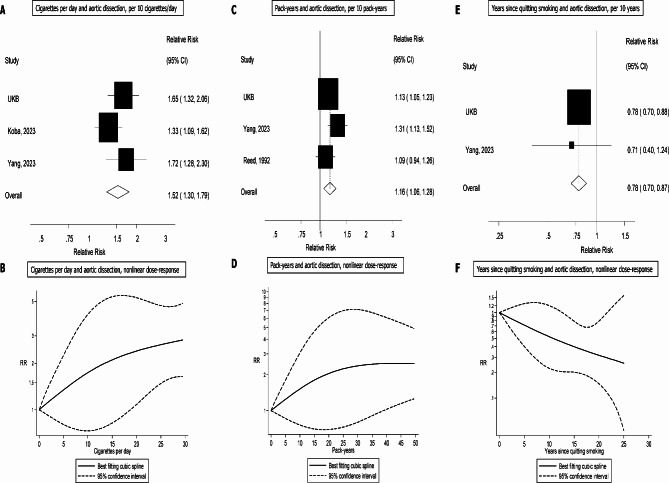
Cigarettes per day, pack-years and years since quitting smoking and aortic dissection.

The literature search identified 5 previously published cohort studies (6 publications)^3,4,12–14,22^ in addition to the current study in UKB, which were included in the systematic review (Fig. 1). Five studies (734757 participants, 782 aortic dissection cases) were included in the meta-analysis of current smoking and aortic dissection and the summary RR was 2.77 (1.81–4.22, I^2^ = 75.9%) (Fig. 2a). For three studies strictly reporting on current vs. never smokers (674 cases, 685942 participants), the summary RR was 2.44 (1.65–3.60, I^2^ = 68%) (Fig. 2b), for former vs. never smokers the summary RR was 1.32 (0.72–2.40, I^2^ = 75%) (Fig. 2c), and for ever vs. never smokers the summary RR was 1.76 (0.98–3.17, I^2^ = 91%) (Fig. 2d). The summary RR per 10 cigarettes/day was 1.52 (1.30–1.79, I^2^ = 31%, *n* = 3) (Fig. 3a) and there was no evidence of nonlinearity (p_nonlinearity_=0.64) (Fig. 3b, Supplemental Table 8). The summary RR per 10 pack-years was 1.16 (1.06–1.28, I^2^ = 44%, *n* = 3) (Fig. 3c) and there was no indication of nonlinearity (p_nonlinearity_=0.46) (Fig. 3d, Supplemental Table 8). The summary RR per 10 year increase since smoking cessation was 0.78 (0.71–0.86, I^2^ = 0%, *n* = 2) (Fig. 3e) and there was no evidence of nonlinearity (p_nonlinearity_=0.89) (Fig. 3f, Supplemental Table 8).

Analyses restricted to studies on aortic dissection mortality gave similar results (Supplemental Figs. 1 and 2). For three studies on smoking status and aortic dissection mortality (458 aortic dissection deaths, 685942 participants), the summary RRs were 2.37 (1.50–3.75, I^2^ = 68%, *n* = 3) for current vs. never smokers, 1.38 (0.77–2.47, I^2^ = 69%, *n* = 3) for former vs. never smokers, and 1.80 (0.99–3.26, I^2^ = 88%, *n* = 3) for ever vs. never smokers (Supplemental Fig. 1), respectively, and 1.56 (1.27–1.92, I^2^ = 46%, *n* = 3) per 10 cigarettes/day (Supplemental Fig. 2a) and there was no evidence of nonlinearity (Supplemental Fig. 2b, Supplemental Table 9). For two studies (270 aortic dissection deaths, 590219 participants) on pack-years and years since smoking cessation, the summary RR was 1.19 (0.99–1.43, I^2^ = 72%, *n* = 2) per 10 pack-years, and 0.85 (0.74–0.97, I^2^ = 0%, *n* = 2) per 10 years of smoking cessation when compared to current smokers (Supplemental Fig. 2c, 2e) and there was no evidence of nonlinearity in either of these analyses (Supplemental Fig. 2d, 2f, Supplemental Table 9).

## Discussion

In this analysis of 0.5 million participants in the UK Biobank study, we found that current smoking was associated with a 2.4-fold increase in risk of aortic dissection, but no clear increase in risk was observed among former smokers. There was also a 65% increased risk of aortic dissection with ≥ 20 pack-years smoked and a 2.5-fold increase among participants smoking 20 or more cigarettes per day compared to never smokers. Younger age at starting smoking was not more strongly associated with risk of aortic dissection than older age (< 16 vs. ≥16 years). Compared to current smokers, risk was reduced 48–75% among former smokers, with some suggestion of stronger reduction in risk with longer duration of smoking cessation, and the risk was comparable to that of never smokers after 10-<20 years of smoking cessation. In a meta-analysis of three cohort studies, a 2–4 to 2.8-fold increase in risk of aortic dissection was observed among current smokers, but there was no clear association among former smokers. There was some evidence of a dose-response relationship between increasing number of cigarettes per day and pack-years and aortic dissection risk, while there was an inverse dose-response relationship between years since quitting and aortic dissection risk. Results were similar in analyses of aortic dissection mortality, but somewhat stronger in sensitivity analyses when additionally excluding participants with prevalent ischemic heart disease, stroke, cancer, or respiratory disease at baseline.

The current results are consistent with the results of the few previous cohort studies and one case-control study which have been published on this association^3,4,12–15^. However, the previous studies had a limited number of cases (23–180 cases), and some adjusted for a limited number of confounding factors, and most studies typically only assessed one measure of smoking, and did not report as comprehensively across different smoking variables as in the current analysis. In addition, some previous studies analysed never and former smokers combined as non-current smokers in the reference category^3,13^, which left the question of whether former smokers also were at increased risk compared to never smokers open. Given that risk of aortic dissection was reduced soon after quitting smoking it seems likely that the damage done by tobacco smoking is relatively quickly reversible and might suggest that tobacco smoking might operate through mechanisms that act proximal in time in relation to the disease development. The findings are also consistent with a previous meta-analysis on smoking and risk of aortic aneurysm^11^, a condition with overlapping risk factors as for aortic dissection.

As with any observational study we cannot rule out residual confounding as tobacco smokers tend to have other unhealthy behaviours which potentially could influence the association between smoking and aortic dissection. However, we adjusted for known or suspected risk factors for aortic dissection, and there was little impact of such adjustments on the results. Considering this as well as the relatively strong associations (2.4–2.8 fold increase in risk) observed it seems less likely that residual confounding fully explains the observed associations. Measurement errors in the assessment of tobacco smoking and changes in smoking habits after baseline may have affected the results, however, because of the prospective design of the current study this would most likely have been non-differential and led to underestimation of the observed associations. Similarly, any misclassification of aortic dissection diagnoses would most likely lead to an underestimation of the true underlying associations. It is possible that participants may have changed their smoking habits due to other chronic diseases or undiagnosed disease. In further sensitivity analyses excluding participants with prevalent ischemic heart disease, stroke, cancer and respiratory disease at baseline, the observed associations were strengthened, but additional exclusion of the first 5 years of follow-up did not substantially alter the results further. Although the UK Biobank is not fully representative of the UK population, the exposure-outcome association is likely to be valid. The findings of the UK Biobank data are most generalisable to middle-aged (37–73 years) Caucasian individuals, however, given that we observed similar results in the meta-analysis, which included studies from the UK, US, and Japan, it is possible that the meta-analytic results could be more widely generalisable.

Strengths of the current analysis include the large sample size and moderately long follow-up (0.5 million participants and > 12 years follow-up) which provided sufficient statistical power to detect associations with a rare outcome like aortic dissection. In addition, the comprehensive assessment of tobacco smoking and covariates allowed for detailed analyses of smoking status, intensity, duration, pack-years, age at starting smoking, and time since quitting smoking and risk of aortic dissection with adjustment for known and potential risk factors for the disease. Detailed subgroup analyses stratified by other risk factors and sensitivity analyses excluding early follow-up and participants with other prevalent cardiovascular diseases, cancer and respiratory disease at baseline were carried out and in general supported the overall results.

The strong dose-response relationship between increasing number of cigarettes smoked and risk of aortic dissection suggests that there may be an underlying biological relationship between the two. Smoking is also strongly associated with increased risk of aortic aneurysms^11^, a closely related disease with shared risk factors^14,23^. Aortic dissection is characterized by degeneration of the aorta media, which consists of a lamellar unit with smooth muscle cells, elastin and collagen. Cigarette smoking increases macrophage production of matrix metalloproteinase and downregulates tissue inhibitor of metalloproteases-3 (TIMP-3)^24^, causing degeneration of the elastin and collagen^25–27^, elastin-specific autoimmunity^28^ and has also been shown to disrupt collagen synthesis^29^. Nicotine has been shown to have similar effects in experimental models, showing clustering of macrophages, neutrophils, and T-cells in areas with structural damage, increased metalloproteinase production, and pathogenic angiogenesis with attenuated fibrosis in the adventitia^30^. Cigarette smoking affects the nourishment of the aorta wall by obstruction of blood flow in small arteries such as the vasa vasorum, contributing to the development of aortic dissection^14^. An experimental study found nicotine reduced collagen levels in vasa vasorum, and may contribute to stenosis of vasa vasorum by inducing abnormal proliferation of smooth muscle cells^31^. Tobacco smoking has also been associated with a small increase in risk of hypertension^17,18^, which is a strong risk factor for aortic dissection^2^, however, this increase in hypertension risk is insufficient to explain the much stronger association between smoking and aortic dissection. In addition, the observed association was not materially altered by adjustment for baseline hypertension status. Some studies have linked smoking with increased risk of reoperation after aortic surgery^32,33^, which could increase risk of complications and mortality. Smoking associated with increased aortic size^34,35^, which predicts worse outcomes^36^, and may contribute to aortic dissection mortality.

## Conclusion

In summary we found a 2.4-fold increased risk of aortic dissection among current smokers in the UK Biobank and in a meta-analysis of cohort studies and there was some suggestion of a dose-response relationship between increasing number of cigarettes smoked per day and pack-years and aortic dissection risk. In contrast, there was no increase in risk among former smokers, and across all durations of smoking cessation a reduced risk was observed when compared to current smokers. These findings add to a long list of tobacco-related disorders and provide further support for interventions and policies for smoking prevention and cessation.

## Electronic supplementary material

Below is the link to the electronic supplementary material.

Supplementary Material 1

Supplementary Material 2

## Data Availability

The data are available through the UK Biobank resource: https://www.ukbiobank.ac.uk/. These data are not available for sharing publicly, but require applications for data access.
