# Supplementary material for: Tobacco smoking and the risk of aortic dissection in the UK Biobank and a meta-analysis of prospective studies
Source: Sci Rep. 2025 Apr 9;15:12083. doi: 10.1038/s41598-025-96529-y (PMC11982237; doi:10.1038/s41598-025-96529-y)

Supplemental Material for

Khan MYI, Dillman A, Sanchez-Perez M, Hibino M, Aune D. Tobacco smoking and the risk of aortic dissection in the UK Biobank and a meta-analysis of prospective studies. Scientific Reports 2025 https://doi.org/10.1038/s41598-025-96529-y

Supplemental Table 1: Hazard ratios (95% CIs) of aortic dissection incidence according to smoking variables - with further adjustment for hypertension status at baseline

Smoking status		Never	Former	Current	Ever			p_{trend}
	Participants	273254	172945	52879	225824			
	Person-years	3391748.3	2112828.2	635847.7	2748675.9			
	Cases	165	137	74	211			
	HR (95% CI)[1]	1.00	1.03 (0.81-1.29)	2.48 (1.87-3.29)	1.29 (1.05-1.59)			
	HR (95% CI)[2]	1.00	1.03 (0.81-1.29)	2.53 (1.90-3.36)	1.29 (1.05-1.59)			
Cigarettes/day		Never	Former	<10 cig/d (5)	10-<20 (14)	≥20 (20)		
	Participants	273254	172945	7207	15405	13477		
	Person-years	3391748.3	2112828.2	87670.2	185514.7	158659.7		
	Cases	165	137	8	23	19		
	HR (95% CI)[1]	1.00	1.03 (0.81-1.29)	2.31 (1.13-4.71)	2.94 (1.88-4.58)	2.63 (1.65-4.37)		<0.001
	HR (95% CI)[2]	1.00	1.03 (0.81-1.29)	2.41 (1.18-4.91)	2.99 (1.92-4.67)	2.70 (1.66-4.40)		<0.001
Pack-years		Never	<10 (5.75)	10-<20 (14.53)	20-<30 (24.25)	≥30 (40.8)		
	Participants	273254	37614	40607	29343	43168		
	Person-years	3391748.3	464066.1	497812.7	356775.1	505817.5		
	Cases	165	30	31	27	58		
	HR (95% CI)[1]	1.00	1.27 (0.86-1.87)	1.12 (0.76-1.66)	1.30 (0.86-1.96)	1.66 (1.21-2.28)		0.002
	HR (95% CI)[2]	1.00	1.28 (0.87-1.89)	1.13 (0.76-1.66)	1.30 (0.86-1.96)	1.66 (1.21-2.28)		0.002
Duration of smoking among current smokers		Never	<30 years (25)	30-<40 (35)	≥40 (45)			
	Participants	273254	8527	13439	16722			
	Person-years	3391748.3	106315.6	164391.7	191617.9			
	Cases	165	9	17	30			
	HR (95% CI)[1]	1.00	3.85 (1.89-7.87)	3.11 (1.84-5.25)	2.19 (1.46-3.30)			<0.001
	HR (95% CI)[2]	1.00	3.91 (1.91-7.97)	3.18 (1.88-5.37)	2.24 (1.49-3.37)			<0.001
Duration of smoking among former smokers		Never	<20 years (12)	20-<30 (24)	≥30 (37)			
	Participants	273254	52082	30328	32328			
	Person-years	3391748.3	642146.6	370545.7	382788.6			
	Cases	165	39	14	41			
	HR (95% CI)[1]	1.00	1.01 (0.71-1.43)	0.59 (0.34-1.02)	1.41 (0.98-2.01)			0.38

	HR (95% CI)[2]	1.00	1.00 (0.70-1.42)	0.58 (0.34-1.01)	1.42 (0.99-2.03)			0.37
Age started smoking		Never	Former, ≤15 years	Former, ≥16 years	Current, ≤15 years	Current, ≥16 years		
	Participants	273254	33930	81268	13204	25484		
	Person-years	3391748.3	410832.9	990133.4	156298.9	306026.3		
	Cases	165	28	67	13	43		
	HR (95% CI)[1]	1.00	1.04 (0.69-1.57)	1.01 (0.75-1.34)	1.88 (1.05-3.35)	3.07 (2.18-4.33)		
	HR (95% CI)[2]	1.00	1.04 (0.69-1.58)	1.00 (0.75-1.34)	1.90 (1.07-3.40)	3.14 (2.23-4.43)		
Years since quitting		Current	<10 years (4)	10-<20 (15)	20-<30 (25)	≥30 (34)	Never	
	Participants	52879	31565	26828	30788	26088	273254	
	Person-years	635847.7	381796.8	326269.6	376698.6	317047.4	3391748.3	
	Cases	74	27	19	16	33	165	
	HR (95% CI)[1]	1.00	0.52 (0.34-0.82)	0.38 (0.23-0.62)	0.25 (0.14-0.43)	0.49 (0.32-0.74)	0.40 (0.30-0.53)	<0.001
	HR (95% CI)[2]	1.00	0.51 (0.33-0.80)	0.37 (0.22-0.61)	0.24 (0.14-0.41)	0.47 (0.31-0.72)	0.39 (0.29-0.52)	<0.001

[1]Model 1: Age, sex, ethnicity, Townsend deprivation index, education, BMI, height, total physical activity, history of connective tissue disease, and each smoking variable.
[2]Model 2: model 1 + hypertension

Supplemental Table 2: Hazard ratios (95% CIs) of aortic dissection mortality according to smoking variables

Smoking status		Never	Former	Current	Ever			p_{trend}
	Participants	273254	172945	52879	225824			
	Person-years	3392172.4	2113126.1	636049.1	2749175.2			
	Deaths	70	63	27	90			
	HR (95% CI)[1]	1.00	1.14 (0.80-1.61)	2.32 (1.47-3.66)	1.34 (0.97-1.84)			
	HR (95% CI)[2]	1.00	1.14 (0.80-1.61)	2.38 (1.51-3.76)	1.43 (0.97-2.12)			
Cigarettes/day	Cut-off (median)	Never	Former	<10 cig/d (5)	10-<20 (14)	≥20 (20)		
	Participants	273254	172945	7207	15405	13477		
	Person-years	3392172.4	2113126.1	87699	185570.3	158720.1		
	Deaths	70	63	2	8	8		
	HR (95% CI)[1]	1.00	1.14 (0.81-1.62)	1.40 (0.34-5.72)	2.63 (1.25-5.52)	3.05 (1.44-6.46)		<0.001
	HR (95% CI)[2]	1.00	1.14 (0.81-1.62)	1.47 (0.36-6.00)	2.69 (1.28-5.65)	3.07 (1.45-6.52)		<0.001
Pack-years	Cut-off (median)	Never	<10 (5.75)	10-<20 (14.53)	20-<30 (24.25)	≥30 (40.8)		
	Participants	273254	37614	40607	29343	43168		
	Person-years	3392172.4	464124.5	497883.9	356848.2	505965.7		
	Deaths	70	18	11	12	21		
	HR (95% CI)[1]	1.00	1.81 (1.08-3.04)	0.97 (0.51-1.83)	1.41 (0.76-2.61)	1.48 (0.89-2.47)		0.15
	HR (95% CI)[2]	1.00	1.83 (1.09-3.08)	0.97 (0.51-1.84)	1.41 (0.76-2.61)	1.49 (0.89-2.48)		0.15
Duration of smoking among current smokers	Cut-off (median)	Never	<30 years (25)	30-<40 (35)	≥40 (45)			
	Participants	273254	8527	13439	16722			
	Person-years	3392172.4	106332.9	164435.6	191707.5			
	Deaths	70	4	4	13			
	HR (95% CI)[1]	1.00	4.20 (1.43-12.31)	1.76 (0.62-4.97)	2.23 (1.20-4.15)			0.003
	HR (95% CI)[2]	1.00	4.25 (1.45-12.46)	1.80 (0.64-5.11)	2.29 (1.23-4.27)			0.002
Duration of smoking among former smokers	Cut-off (median)	Never	<20 years (12)	20-<30 (24)	≥30 (37)			
	Participants	273254	52082	30328	32313			
	Person-years	3392172.4	642227	370575	382881.1			
	Deaths	70	22	8	15			
	HR (95% CI)[1]	1.00	1.36 (0.84-2.21)	0.81 (0.39-1.70)	1.22 (0.69-2.18)			0.61
	HR (95% CI)[2]	1.00	1.36 (0.84-2.21)	0.80 (0.38-1.69)	1.23 (0.69-2.19)			0.61

Age started smoking	Cut-off	Never	Former, ≤15 years	Former, ≥16 years	Current, ≤15 years	Current, ≥16 years		
	Participants	273254	33930	81268	13204	25484		
	Person-years	3392172.4	410874.1	990296.1	156341.8	306134.3		
	Deaths	70	11	34	7	14		
	HR (95% CI)[1]	1.00	1.09 (0.57-2.09)	1.22 (0.80-1.85)	2.85 (1.28-6.34)	2.55 (1.42-4.57)		
	HR (95% CI)[2]	1.00	1.09 (0.57-2.10)	1.22 (0.80-1.85)	2.92 (1.31-6.50)	2.62 (1.46-4.70)		
Years since quitting	Cut-off (median)	Current	<10 years (4)	10-<20 (15)	20-<30 (25)	≥30 (34)	Never	
	Participants	52879	31565	26828	30788	26088	273254	
	Person-years	636049.1	381864.3	326302.0	376736.6	317111.7	3392172.4	
	Deaths	27	9	9	7	21	70	
	HR (95% CI)[1]	1.00	0.46 (0.22-0.99)	0.47 (0.22-1.00)	0.28 (0.12-0.65)	0.80 (0.27-1.44)	0.43 (0.27-0.68)	0.28
	HR (95% CI)[2]	1.00	0.46 (0.21-0.97)	0.45 (0.21-0.97)	0.27 (0.12-0.63)	0.78 (0.43-1.40)	0.42 (0.26-0.66)	0.25

[1]Model 1: Age, sex, ethnicity, Townsend deprivation index, education, BMI, height, total physical activity, history of connective tissue disease, and each smoking variable.

[2]Model 2: model 1 + hypertension

Supplemental Table 3: Hazard ratios (95% CIs) of aortic dissection incidence according to smoking variables, sensitivity analysis excluding participants with prevalent ischemic heart disease, stroke, cancer, and respiratory disease at baseline

Smoking status		Never	Former	Current	Ever			p_{trend}
	Participants	204758	118522	36979	155501			
	Person-years	2554529.1	1465210.1	451935.2	1917145.3			
	Cases	101	87	48	135			
	HR (95% CI)	1.00	1.20 (0.89-1.60)	3.02 (2.12-4.30)	1.52 (1.17-1.98)			
Cigarettes/day		Never	Former	<10 cig/d (5)	10-<20 (14)	≥20 (20)		
	Participants	204758	118522	5292	10670	8873		
	Person-years	2554529.1	1465210.1	65117.3	130458.3	106724.3		
	Cases	101	87	6	13	16		
	HR (95% CI)	1.00	1.19 (0.89-1.59)	2.98 (1.31-6.82)	3.21 (1.79-5.77)	4.61 (2.68-7.95)		<0.001
Pack-years		Never	<10 (5.75)	10-<20 (14.53)	20-<30 (24.25)	≥30 (40.8)		
	Participants	204758	27693	28954	19613	25168		
	Person-years	2554529.1	343817.4	358171.5	241442.5	302523.1		
	Cases	101	19	21	14	38		
	HR (95% CI)	1.00	1.33 (0.82-2.18)	1.33 (0.83-2.14)	1.29 (0.74-2.28)	2.39 (1.62-3.54)		<0.001
Duration of smoking among current smokers		Never	<30 years (25)	30-<40 (35)	≥40 (45)			
	Participants	205758	6547	9605	10888			
	Person-years	2554529.1	81983.6	118636.7	124032			
	Cases	101	5	15	17			
	HR (95% CI)	1.00	4.39 (1.70-11.35)	5.58 (3.13-9.93)	2.46 (1.44-4.19)			<0.001
Duration of smoking among former smokers		Never	<20 years (12)	20-<30 (24)	≥30 (37)			
	Participants	204758	37571	20336	18414			
	Person-years	2554529.1	466435.7	251229.4	223686.9			
	Cases	101	28	6	24			
	HR (95% CI)	1.00	1.22 (0.80-1.86)	0.46 (0.20-1.06)	1.72 (1.08-2.72)			0.19
Age started smoking		Never	Former, ≤15 years	Former, ≥16 years	Current, ≤15 years	Current, ≥16 years		
	Participants	204758	21505	55102	8370	18323		
	Person-years	2554529.1	265004.8	679814.3	101476.3	223176		
	Cases	101	17	41	9	28		

	HR (95% CI)	1.00	1.36 (0.80-2.30)	1.12 (0.77-1.62)	2.91 (1.45-5.84)	3.59 (2.34-5.51)		
Years since quitting		Current	<10 years (4)	10-<20 (15)	20-<30 (25)	≥30 (34)	Never	
	Participants	36979	20191	17433	21184	17877	204758	
	Person-years	451935.2	248915.2	215372.1	262017.3	219463.5	2554529.1	
	Cases	48	17	12	7	23	101	
	HR (95% CI)	1.00	0.57 (0.32-0.99)	0.40 (0.21-0.75)	0.16 (0.07-0.36)	0.48 (0.29-0.81)	0.33 (0.23-0.46)	<0.001

Supplemental Table 4: Hazard ratios (95% CIs) of aortic dissection mortality according to smoking variables, sensitivity analysis excluding participants with prevalent ischemic heart disease, stroke, cancer, and respiratory disease at baseline

Smoking status		Never	Former	Current	Ever			p_{trend}
	Participants	204758	118522	36979	155501			
	Person-years	2554776.8	1465363.5	452068.5	1917432			
	Deaths	48	43	16	59			
	HR (95% CI)	1.00	1.26 (0.83-1.92)	2.21 (1.24-3.94)	1.43 (0.97-2.10)			
Cigarettes/day	Cut-off (median)	Never	Former	<10 cig/d (5)	10-<20 (14)	≥20 (20)		
	Participants	204758	118522	5292	10670	8873		
	Person-years	2554776.8	1465363.5	65133.8	130486.7	106775.5		
	Deaths	48	43	2	3	7		
	HR (95% CI)	1.00	1.25 (0.82-1.91)	2.13 (0.51-8.78)	1.64 (0.51-5.30)	4.54 (2.00-10.27)		0.001
Pack-years	Cut-off (median)	Never	<10 (5.75)	10-<20 (14.53)	20-<30 (24.25)	≥30 (40.8)		
	Participants	204758	27693	28954	19613	25168		
	Person-years	2554776.8	343850.2	358207.8	241468.9	302622.0		
	Deaths	48	13	8	6	15		
	HR (95% CI)	1.00	1.92 (1.04-3.55)	1.08 (0.51-2.30)	1.18 (0.50-2.78)	2.00 (1.09-3.68)		0.05
Duration of smoking among current smokers	Cut-off (median)	Never	<30 years (25)	30-<40 (35)	≥40 (45)			
	Participants	205758	6547	9605	10541			
	Person-years	2554776.8	81994.9	118670.6	124085.7			
	Deaths	48	3	3	7			
	HR (95% CI)	1.00	3.38 (1.29-15.60)	2.00 (0.60-6.65)	1.98 (0.87-4.51)			0.03
Duration of smoking among former smokers	Cut-off (median)	Never	<20 years (12)	20-<30 (24)	≥30 (37)			
	Participants	204758	37571	20336	18414			
	Person-years	2554776.8	466478.1	251232	223740.5			
	Deaths	48	18	4	10			
	HR (95% CI)	1.00	1.66 (0.96-2.87)	0.66 (0.23-1.84)	1.50 (0.75-3.02)			0.38
Age started smoking	Cut-off	Never	Former, ≤15 years	Former, ≥16 years	Current, ≤15 years	Current, ≥16 years		
	Participants	204758	21505	55102	8370	18323		
	Person-years	2554776.8	265015.2	679902.4	101495.8	223255.4		
	Deaths	48	10	22	5	8		

	HR (95% CI)	1.00	1.85 (0.91-3.73)	1.28 (0.76-2.13)	3.71 (1.44-9.57)	2.23 (1.05-4.77)		
Years since quitting	Cut-off (median)	Current	<10 years (4)	10-<20 (15)	20-<30 (25)	≥30 (34)	Never	
	Participants	36979	20191	17433	21184	17877	204758	
	Person-years	452068.5	248968.4	215380.0	262024.4	219494	2554776.8	
	Deaths	16	6	6	4	17	48	
	HR (95% CI)	1.00	0.59 (0.23-1.52)	0.60 (0.23-1.53)	0.28 (0.09-0.84)	1.07 (0.53-2.17)	0.45 (0.25-0.81)	0.89

Supplemental Table 5: Hazard ratios (95% CIs) of aortic dissection incidence according to smoking variables, sensitivity analysis excluding participants with prevalent ischemic heart disease, stroke, cancer, and respiratory disease at baseline and first 5 years of follow-up

Smoking status		Never	Former	Current	Ever			p_{trend}
	Participants	202773	116797	36121	152918			
	Person-years	1534504.0	876024.3	268813.5	1144837.8			
	Cases	77	66	37	103			
	HR (95% CI)	1.00	1.20 (0.86-1.68)	3.10 (2.07-4.64)	1.54 (1.14-2.09)			
Cigarettes/day		Never	Former	<10 cig/d (5)	10-<20 (14)	≥20 (20)		
	Participants	202773	116797	5206	10436	8555		
	Person-years	1534504.0	876024.3	38842.7	77593.7	63009.4		
	Cases	77	66	5	11	11		
	HR (95% CI)	1.00	1.19 (0.85-1.67)	3.31 (1.33-8.20)	3.60 (1.89-6.83)	4.26 (2.23-8.17)		<0.001
Pack-years		Never	<10 (5.75)	10-<20 (14.53)	20-<30 (24.25)	≥30 (40.8)		
	Participants	202773	27392	28550	19274	24355		
	Person-years	1534504.0	205952.2	214199.4	144111.9	178309.6		
	Cases	77	15	18	11	26		
	HR (95% CI)	1.00	1.39 (0.80-2.43)	1.51 (0.90-2.54)	1.34 (0.71-2.54)	2.18 (1.37-3.48)		0.001
Duration of smoking among current smokers		Never	<30 years (25)	30-<40 (35)	≥40 (45)			
	Participants	202773	6476	9416	10097			
	Person-years	1534504.0	49415.6	71030.7	72194.8			
	Cases	77	5	11	12			
	HR (95% CI)	1.00	5.61 (2.14-14.69)	5.24 (2.68-10.21)	2.28 (1.22-4.28)			<0.001
Duration of smoking among former smokers		Never	<20 years (12)	20-<30 (24)	≥30 (37)			
	Participants	202773	37133	20020	17945			
	Person-years	1534504.0	279445.7	150184.9	132565.3			
	Cases	77	21	6	17			
	HR (95% CI)	1.00	1.22 (0.75-1.99)	0.62 (0.27-1.44)	1.65 (0.96-2.84)			0.25
Age started smoking		Never	Former, ≤15 years	Former, ≥16 years	Current, ≤15 years	Current, ≥16 years		
	Participants	202773	21137	54242	8126	17863		
	Person-years	1534504.0	158197.4	406045	60104.5	132536.5		
	Cases	77	11	33	7	21		

	HR (95% CI)	1.00	1.16 (0.61-2.21)	1.19 (0.78-1.80)	3.07 (1.39-6.76)	3.63 (2.22-5.93)		
Years since quitting		Current	<10 years (4)	10-<20 (15)	20-<30 (25)	≥30 (34)	Never	
	Participants	36121	19816	17157	20873	17607	202773	
	Person-years	268813.5	148710.6	128760.3	156732.6	130609	1534504.0	
	Cases	37	12	11	5	17	77	
	HR (95% CI)	1.00	0.51 (0.26-0.98)	0.47 (0.24-0.92)	0.15 (0.06-0.38)	0.46 (0.26-0.84)	0.32 (0.21-0.48)	0.002

Supplemental Table 6: Hazard ratios (95% CIs) of aortic dissection mortality according to smoking variables, sensitivity analysis excluding participants with prevalent ischemic heart disease, stroke, cancer, and respiratory disease at baseline and first 5 years of follow-up

Smoking status		Never	Former	Current	Ever			p_{trend}
	Participants	202773	116797	36121	152918			
	Person-years	1534728.0	876157.5	268927.2	1145084.7			
	Deaths	38	36	12	48			
	HR (95% CI)	1.00	1.36 (0.85-2.16)	2.17 (1.12-4.21)	1.50 (0.97-2.31)			
Cigarettes/day	Cut-off (median)	Never	Former	<10 cig/d (5)	10-<20 (14)	≥20 (20)		
	Participants	202773	116797	5206	10436	8555		
	Person-years	1534728.0	876157.5	38859.2	77619.6	63053.0		
	Deaths	38	36	1	2	6		
	HR (95% CI)	1.00	1.36 (0.85-2.16)	1.35 (0.18-9.85)	1.39 (0.33-5.81)	5.12 (2.11-12.45)		0.003
Pack-years	Cut-off (median)	Never	<10 (5.75)	10-<20 (14.53)	20-<30 (24.25)	≥30 (40.8)		
	Participants	202773	27392	28550	19274	24355		
	Person-years	1534728.0	205980.3	214234.2	144136.9	178393.6		
	Deaths	38	11	7	4	12		
	HR (95% CI)	1.00	2.08 (1.06-4.08)	1.22 (0.54-2.74)	1.02 (0.36-2.89)	2.14 (1.09-4.22)		0.06
Duration of smoking among current smokers	Cut-off (median)	Never	<30 years (25)	30-<40 (35)	≥40 (45)			
	Participants	202733	6476	9416	10097			
	Person-years	1534728.0	49426.9	71061.8	72241.2			
	Deaths	38	3	1	5			
	HR (95% CI)	1.00	5.68 (1.59-20.38)	0.85 (0.11-6.34)	1.83 (0.70-4.80)			0.13
Duration of smoking among former smokers	Cut-off (median)	Never	<20 years (12)	20-<30 (24)	≥30 (37)			
	Participants	202733	37133	20020	17945			
	Person-years	1534728.0	279482	150187.6	132612.7			
	Deaths	38	15	4	8			
	HR (95% CI)	1.00	1.80 (0.98-3.31)	0.87 (0.31-2.47)	1.63 (0.74-3.56)			0.25
Age started smoking	Cut-off	Never	Former, ≤15 years	Former, ≥16 years	Current, ≤15 years	Current, ≥16 years		
	Participants	202735	21130	95236	8123	27646		
	Person-years	1534728.0	158204.6	406124.1	60124.1	132605.8		
	Deaths	38	7	20	3	5		

	HR (95% CI)	1.00	1.67 (0.73-3.82)	1.50 (0.86-2.60)	2.94 (0.88-9.76)	2.19 (0.92-5.24)		
Years since quitting	Cut-off (median)	Current	<10 years (4)	10-<20 (15)	20-<30 (25)	≥30 (34)	Never	
	Participants	36110	19811	17151	20870	58929	202735	
	Person-years	268927.2	148757.6	128768.2	156738.6	130634.5	1534728.0	
	Deaths	12	5	6	3	22	38	
	HR (95% CI)	1.00	0.66 (0.23-1.87)	0.79 (0.29-2.11)	0.27 (0.08-0.98)	1.15 (0.52-2.54)	0.46 (0.24-0.89)	0.90

Supplemental Table 7: Analyses of smoking status and aortic dissection, stratified by age, sex, BMI categories and hypertension status

Smoking status			Never	Former	Current	$P_{interaction}$
Age	<60 years	HR (95% CI)	1.00	0.65 (0.41-1.02)	1.77 (1.12-2.79)	0.07
	≥60 years	HR (95% CI)	1.00	1.28 (0.97-1.69)	3.07 (2.14-4.41)	
Sex	Men	HR (95% CI)	1.00	0.97 (0.72-1.31)	2.22 (1.55-3.18)	0.73
	Women	HR (95% CI)	1.00	1.13 (0.78-1.63)	3.03 (1.91-4.82)	
BMI	<25	HR (95% CI)	1.00	1.23 (0.78-1.94)	2.02 (1.12-3.64)	0.44
	25-<30	HR (95% CI)	1.00	0.91 (0.65-1.28)	2.26 (1.46-3.48)	
	≥30	HR (95% CI)	1.00	1.06 (0.68-1.64)	3.15 (1.89-5.22)	
Hypertension	No	HR (95% CI)	1.00	1.13 (0.65-1.95)	3.43 (1.90-6.18)	0.46
	Yes	HR (95% CI)	1.00	1.00 (0.78-1.29)	2.29 (1.65-3.17)	

Supplemental Table 8. Prospective studies of smoking and aortic dissection

First author, publication year, country	Study name or description	Study period	Number of participants, number of cases	Smoking exposure	Comparison	Relative risk (95% confidence interval)	Adjustment for confounders
Strachan DP, 1991, United Kingdom	The Whitehall Study	1967-1969 - 1987, 18 years follow-up	18403 men, age 40-64 years: 38 aortic dissection deaths	Manufactured cigarette smoking<br>Hand-rolled cigarette smoking<br>Pipe, cigar smoking	Never/former smoker<br>Current<br>Never/former smoker<br>Current<br>Never/former smoker<br>Current	1.0<br>16.7 (3.4-82.1)<br>1.0<br>7.7 (2.8-21.7)<br>1.0<br>56.5 (13.0-246)	Age
Reed D et al, 1992, USA	Honolulu Heart Program	1965-1968 - 1988, 20 years follow-up	7682 men, age 46-68 years: 23 aortic dissection cases	Cigarette pack-years	Per 62 pack-years	1.68 (0.67-4.18)	Age, systolic blood pressure, sercum cholesterol, serum triglycerides, height
Landenhed M et al, 2017, Sweden	Malmo Diet and Cancer Study	1991-1996 - NA, 16 years follow-up	30412 men and women, age 41-73 years: 70 aortic dissection cases	Smoking	Regular/cessation last year vs. non-smoker/cessation for more than one year	1.91 (1.12-3.25)	Age, sex, hypertension, apoA1
Kihara T et al, 2017, Japan	Japan Collaborative Cohort Study	1988-1990 - 2009, 19 years follow-up	48677 men and women, age 40-79 years: 66 aortic dissection deaths	Passive smoking among never smokers<br><br><br><br><br>Passive smoking at home<br><br><br><br><br>Combination of passive smoking	Low<br>Intermediate<br>High<br>Former smokers<br>Current smokers<br>Low<br>Intermediate<br>High<br>Former smokers<br>Current smokers<br>Low<br>Intermediate<br>High<br>Former smokers<br>Current smokers	1.00<br>1.31 (0.55-3.15)<br>1.78 (0.56-5.61)<br>1.66 (0.57-4.87)<br>3.39 (1.43-8.00)<br>1.00<br>0.69 (0.25-1.87)<br>2.33 (0.95-5.70)<br>1.48 (0.50-4.39)<br>3.03 (1.26-7.29)<br>1.00<br>0.92 (0.33-2.55)<br>2.38 (0.90-6.25)<br>1.76 (0.54-5.77)<br>3.62 (1.33-9.83)	Age, sex, BMI, hypertension, alcohol intake, perceived mental stress, walking, age of completed education, job status, region
Koba A et al, 2023, Japan	Ibaraki Prefectural Health Study	1993 - 2019, 26 years follow-up	95723 men and women, age 40-79 years: 188 aortic dissection deaths	Smoking status	Never<br>Former<br>Current, 1-19 cig/d<br>Current, ≥20	1.00<br>0.89 (0.44-1.80)<br>0.68 (0.30-1.55)<br>2.40 (1.34-4.28)	Age, sex, systolic blood pressure, diastolic blood pressure, HDL cholesterol, non-HDL cholesterol, triglycerides, lipid-lowering medication, serum glucose, alcohol

Yang Y et al, 2023, Japan	Japan Collaborative Cohort Study	1989-1990 - 2009, 16.4 years follow-up	91141 men and women, age 40-79 years: 110 aortic dissection deaths	Smoking status and cigarettes/day	Never	1.00	Age, sex, area, BMI, hypertension, diabetes, drinking status, age when completed education, occupation, perceived mental stress, walking, sports
					Former	2.75 (1.28-5.90)	
					Current, <15 cig/d	2.90 (1.33-6.33)	
					15-24	4.28 (1.62-8.55)	
					≥25	4.07 (1.62-10.21)	
				Pack-years	Never	1.00	
					<20 pack-years	2.86 (1.41-5.80)	
					20-39	4.02 (1.98-8.16)	
					≥40	3.62 (1.67-7.87)	
					Per 10 pack-years	1.04 (0.91-1.19)	
				Years since smoking cessation	Current	1.00	
					0-4 years	0.92 (0.36-2.35)	
					5-9	0.87 (0.31-2.44)	
					10-15	0.84 (0.30-2.38)	
					>15	0.35 (0.08-1.47)	
					Never	0.31 (0.12-0.78)	
					Per 10 years	0.59 (0.28-1.23)	
				Smoking status and cigarettes/day, men	Never	1.00	
					Former	6.89 (1.55-30.53)	
					Current, <15 cig/d	7.80 (1.56-38.89)	
					15-24	9.89 (2.31-42.29)	
					≥25	7.70 (1.58-37.53)	
				Pack-years	Never	1.00	
					<20 pack-years	6.21 (1.28-30.13)	
					20-39	9.50 (2.23-40.38)	
					≥40	7.83 (1.80-34.04)	
					Per 10 pack-years	1.03 (0.89-1.19)	
				Years since smoking cessation	Current	1.00	
					0-4 years	1.11 (0.43-2.86)	
					5-9	0.98 (0.34-2.80)	
					10-15	0.75 (0.23-2.47)	
					>15	0.42 (0.10-1.78)	
					Never	0.16 (0.03-0.74)	
					Per 10 years	0.54 (0.24-1.22)	
				Smoking status and cigarettes/day, women	Never	1.00	
					Former	1.47 (0.20-10.82)	
					Current, <15 cig/d	2.26 (0.70-7.30)	
					15-24	1.46 (0.20-10.73)	
					≥25	9.45 (1.28-69.49)	

				Pack-years	Never	1.00	
					<20 pack-years	2.66 (1.04-6.81)	
					20-39	-	
					≥40	4.43 (0.60-32.88)	
					Per 10 pack-years	1.13 (0.68-1.86)	
				Years since smoking cessation	Current	1.00	
					0-4 years	-	
					5-9	-	
					10-15	2.79 (0.32-24.92)	
					>15	-	
					Never	-	
					Per 10 years	-	
Current study	UK Biobank	2006-2010 - 2021, 12.3 years follow-up	499078 men and women, age 37-73 years: 376 aortic dissection cases<br>160 aortic dissection deaths	Smoking status, aortic dissection incidence	Never	1.00	Age, sex, ethnicity, education, Townsend Deprivation Index, BMI, height, physical activity, connective tissue disease
					Former	1.02 (0.81-1.29)	
					Current	2.48 (1.87-3.29)	
				Cigarettes per day	Never	1.00	
					Former	1.02 (0.81-1.29)	
					<10 cig/d	2.28 (1.12-4.66)	
					10-<20	2.88 (1.85-4.50)	
					≥20	2.62 (1.61-4.27)	
				Pack-years	Never	1.00	
					<10 pack-years	1.26 (0.86-1.87)	
					10-<20	1.12 (0.76-1.64)	
					20-<30	1.28 (0.85-1.93)	
					≥30	1.62 (1.18-2.21)	
				Duration of smoking, current smokers	Never	1.00	
					<30 years	3.86 (1.89-7.89)	
					30-<40	3.11 (1.84-5.26)	
					≥40	2.15 (1.43-3.23)	
				Duration of smoking, former smokers	Never	1.00	
					<20 years	1.08 (0.77-1.52)	
					20-<30	0.57 (0.33-0.99)	
					≥30	1.44 (1.02-2.03)	
				Age started smoking	Never	1.00	
					Former, age ≤15 years	1.03 (0.68-1.55)	
					Former, age ≥16 years	1.02 (0.80-1.30)	
					Current, age ≤15 years	1.82 (1.02-3.23)	
					Current, age ≥16 years	2.71 (2.01-3.66)	
				Years since smoking	Current	1.00	

				cessation	<10 years<br>10-<20<br>20-<30<br>≥30<br>Never	0.53 (0.34-0.83)<br>0.38 (0.23-0.63)<br>0.25 (0.14-0.43)<br>0.45 (0.32-0.63)<br>0.40 (0.30-0.54)	
				Smoking status, aortic dissection mortality	Never<br>Former<br>Current	1.00<br>1.14 (0.80-1.61)<br>2.32 (1.47-3.66)	
				Cigarettes per day	Never<br>Former<br><10 cig/d<br>10-<20<br>≥20	1.00<br>1.14 (0.80-1.61)<br>1.38 (0.34-5.64)<br>2.56 (1.22-5.37)<br>2.95 (1.39-6.24)	
				Pack-years	Never<br><10 pack-years<br>10-<20<br>20-<30<br>≥30	1.00<br>1.81 (1.08-3.04)<br>0.96 (0.51-1.82)<br>1.39 (0.75-2.57)<br>1.43 (0.86-2.38)	
				Duration of smoking, current smokers	Never<br><30 years<br>30-<40<br>≥40	1.00<br>4.21 (1.44-12.33)<br>1.76 (0.62-4.98)<br>2.18 (1.17-4.06)	
				Duration of smoking, former smokers	Never<br><20 years<br>20-<30<br>≥30	1.00<br>1.45 (0.90-2.31)<br>0.79 (0.38-1.66)<br>1.23 (0.70-2.15)	
				Age started smoking	Never<br>Former, age ≤15 years<br>Former, age ≥16 years<br>Current, age ≤15 years<br>Current, age ≥16 years	1.00<br>1.05 (0.55-2.02)<br>1.14 (0.79-1.65)<br>2.69 (1.21-5.96)<br>2.26 (1.37-3.75)	
				Years since smoking cessation	Current<br><10 years<br>10-<20<br>20-<30<br>≥30<br>Never	1.00<br>0.47 (0.22-1.01)<br>0.47 (0.22-1.01)<br>0.29 (0.12-0.66)<br>0.58 (0.35-0.96)<br>0.43 (0.27-0.68)	

apoA1; apolipoprotein A1, BMI; body mass index, HDL; high-density lipoprotein

Supplemental Table 9. Relative risks (95% confidence intervals) from nonlinear analysis of cigarettes/day, pack-years and years since quitting smoking and aortic dissection

Cigarettes/day		Pack-years		Years since quitting smoking	
No	RR (95% CI)	No	RR (95% CI)	No	RR (95% CI)
0	1.00	0	1.00	0	1.00
5	1.35 (0.80-2.25)	5	1.24 (0.86-1.80)	5	0.71 (0.40-1.27)
10	1.74 (0.73-4.13)	10	1.52 (0.76-3.06)	10	0.52 (0.22-1.22)
15	2.10 (0.82-5.36)	15	1.81 (0.70-4.67)	15	0.40 (0.20-0.79)
20	2.39 (1.08-5.29)	20	2.05 (0.69-6.08)	20	0.32 (0.13-0.75)
25	2.63 (1.48-4.69)	25	2.25 (0.73-6.95)	25	0.26 (0.04-1.60)
30	2.82 (1.64-4.86)	30	2.38 (0.80-7.11)	30	0.21 (0.01-4.56)
		35	2.46 (0.90-6.70)		
		40	2.49 (1.04-6.01)		
		45	2.50 (1.17-5.33)		
		50	2.49 (1.26-4.92)		
$p_{nonlinearity}$	0.64	$p_{nonlinearity}$	0.46	$p_{nonlinearity}$	0.89

Supplemental Table 10. Relative risks (95% confidence intervals) from nonlinear analysis of cigarettes/day, pack-years and years since quitting smoking and aortic dissection mortality

Cigarettes/day		Pack-years		Years since quitting smoking	
No	RR (95% CI)	No	RR (95% CI)	No	RR (95% CI)
0	1.00	0	1.00	0	1.00
5	1.34 (0.79-2.27)	5	1.27 (0.89-1.81)	5	0.72 (0.71-0.74)
10	1.73 (0.71-4.22)	10	1.58 (0.81-3.06)	10	0.55 (0.48-0.63)
15	2.13 (0.80-5.66)	15	1.88 (0.75-4.68)	15	0.45 (0.30-0.70)
20	2.50 (1.07-5.83)	20	2.12 (0.73-6.18)	20	0.40 (0.17-0.95)
25	2.85 (1.50-5.41)	25	2.27 (0.72-7.15)	25	0.37 (0.09-1.49)
30	3.17 (1.78-5.64)	30	2.33 (0.72-7.47)	30	0.35 (0.05-2.50)
		35	2.31 (0.73-7.27)		
		40	2.23 (0.74-6.79)		
		45	2.13 (0.72-6.28)		
		50	2.02 (0.69-5.96)		
$p_{nonlinearity}$	0.71	$p_{nonlinearity}$	0.26	$p_{nonlinearity}$	0.36

Supplemental Figure 1. Smoking status and aortic dissection mortality

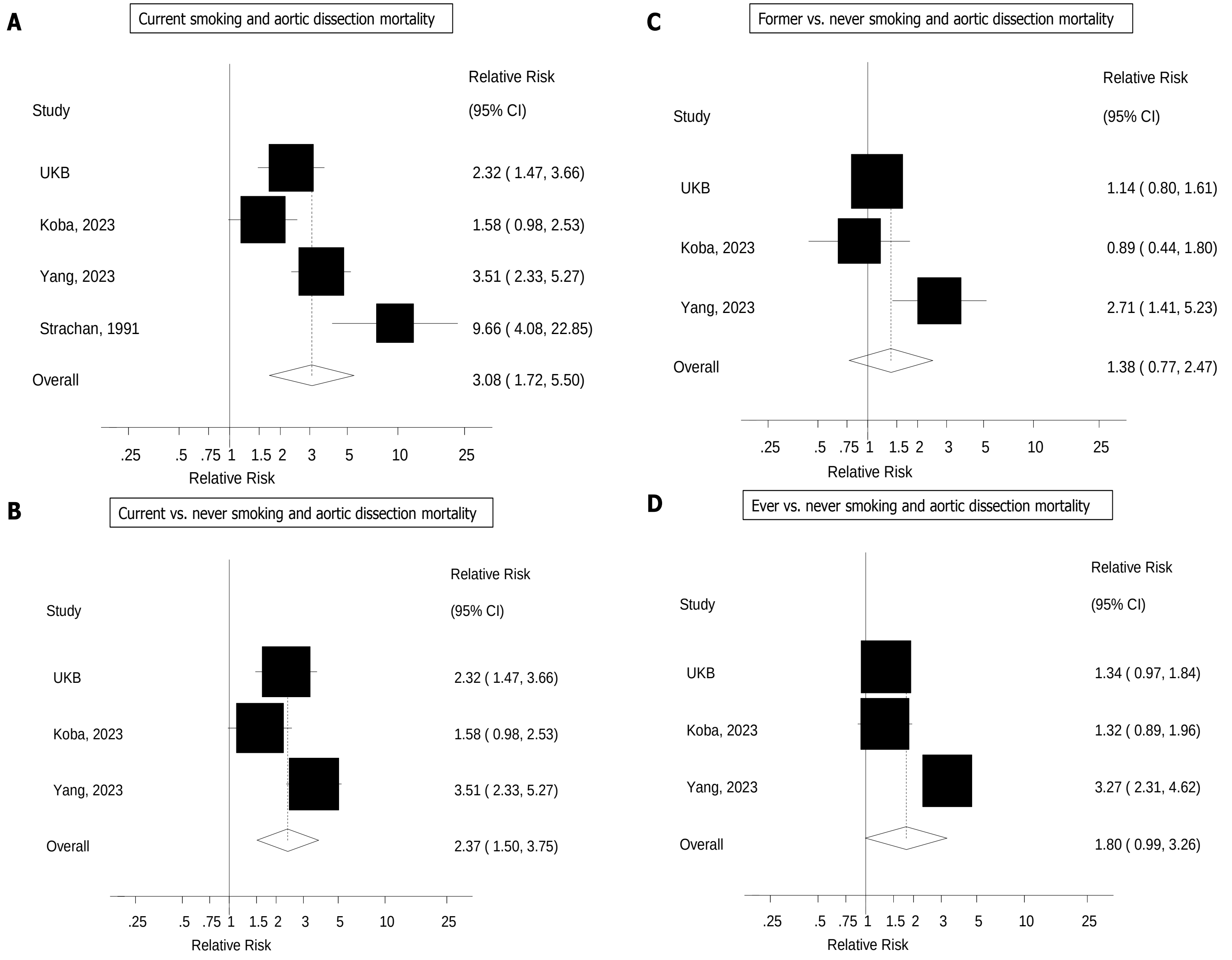

Supplemental Figure 2. Cigarettes per day, pack-years and years since quitting smoking and aortic dissection mortality

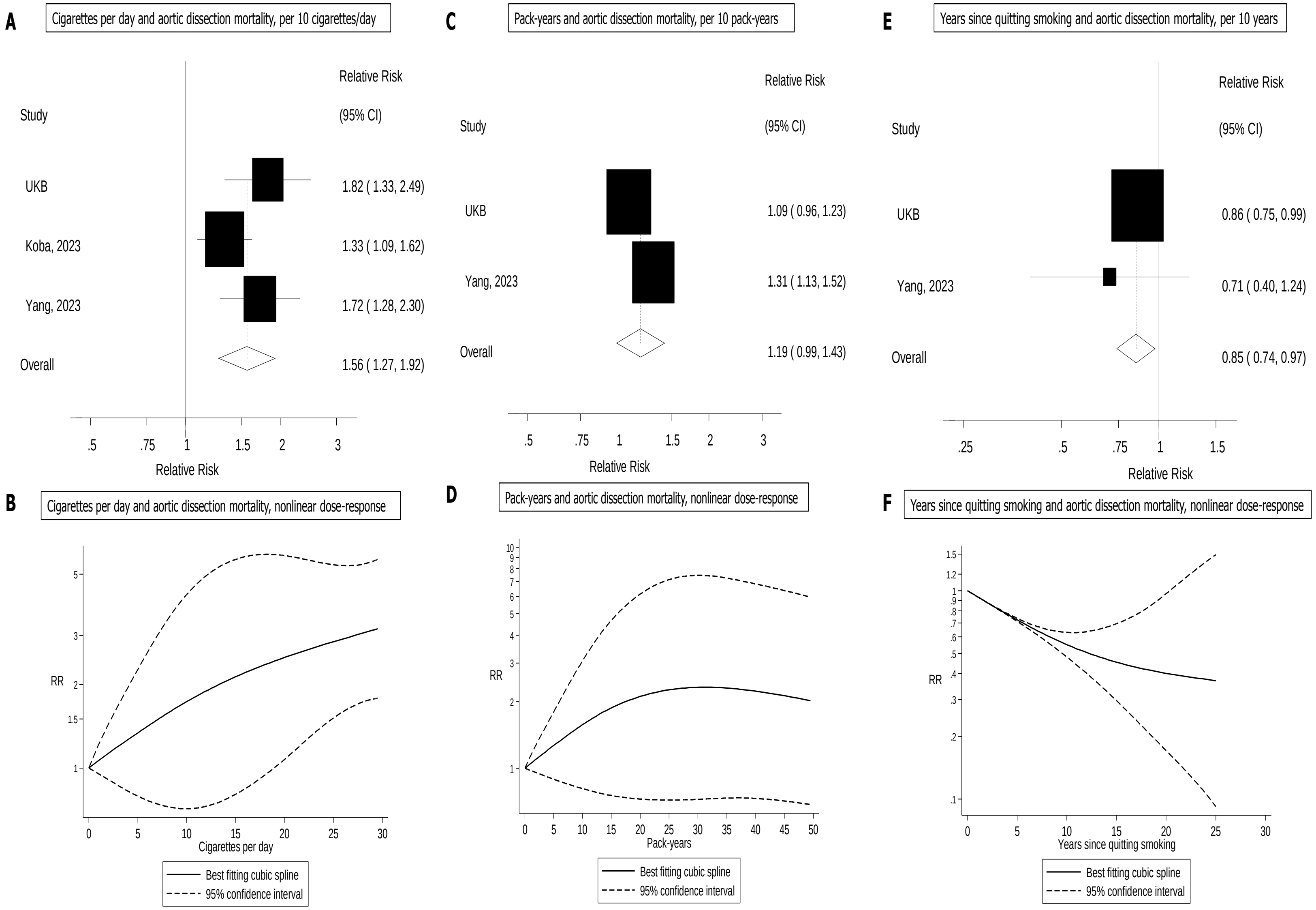

Supplement: Supplementary file 2 — Supplementary Material 2 [file 41598_2025_96529_MOESM2_ESM.pdf]
